# Clinical characterization and outcome of primary bone lymphoma: a retrospective study of 61 Chinese patients

**DOI:** 10.1038/srep28834

**Published:** 2016-06-30

**Authors:** XuanYe Zhang, Jun Zhu, YuQin Song, LingYan Ping, Wen Zheng

**Affiliations:** 1 Department of Lymphoma, Peking University Cancer Hospital & Institute, 52 Fucheng Road, Beijing 100142, China; 2 Department of Medical Oncology, Sun Yat-Sen University Cancer Center, 651 Dongfeng East Road, Guangzhou 510060, China

## Abstract

Primary bone lymphoma(PBL) is a rare disease. To assess the clinical characteristics, outcome, and prognostic factors of this entity in Chinese population, we retrospectively analyzed 61 PBL patients initially treated in our institution between 1997 and 2014. The median age was 45 years. The most common histological subtype was diffuse large B-cell lymphoma (DLBCL) (55.7%), followed by T-cell lymphoma (18.0%). All patients underwent systemic chemotherapy as initial treatment while 24 patients (39.3%) were additionally treated with radiotherapy. The 5-year overall survival (OS) and the 5-year progression-free survival (PFS) rates of 57 cases with completed follow-up were 52.3% and 40.1%, respectively. In further analysis of the primary bone DLBCL (PB-DLBCL) subgroup, the 5-year OS and PFS rates were 53.0% and 47.0%, and a multivariable analysis revealed that baseline Eastern Cooperative Oncology Group (ECOG) score and response to initial treatment (complete remission versus no complete remission) were independent prognostic factors for both OS and PFS. The proportion of T-cell lymphoma is higher in China than in western populations. High baseline ECOG scores (≥2) and unachieved CR in initial therapy were factors for poor PB-DLBCL prognosis. The role of radiotherapy and rituximab in PLB therapy remains to be confirmed in further investigation.

Primary bone lymphoma (PBL) is a rare primary extranodal lymphoma in the skeletal system that accounts for less than 1% of all lymphomas, about 4–5% of extranodal lymphomas, and about 3% of all malignant bone tumors^1^,^2^,^3^. The definition of PBL was controversial in the past, but has been defined in the new version of “WHO pathology and genetics classification of soft tissue and bone tumor”^4^ in 2013 as: *a kind of malignant tumor composed by malignant lymphocytes, forming single or multiple tumor in the bone, not associated with infringement or violation of other extranodal malignant lymph nodes outside the area.* The disease can occur at any age, with a median age of onset ranging from 40–60 years old, with most of the literature suggesting that the proportion in male patients was slightly higher than in females (1.0–1.8:1), a finding also reported for children^5^,^6^. Most PBL cases belong to B cell non-Hodgkin’s lymphoma, the most common type being diffuse large B-cell lymphoma (DLBCL)^7^,^8^,^9^. Other types include follicular lymphoma, mantle cell lymphoma, B-lymphoblastic lymphoma, small lyphocytic lymphoma and Burkitt lymphoma. PBL’s clinical manifestations are not specific but the most common symptoms are pain and mass^10^,^11^,^12^,^13^. PBL can occur in various parts of bone tissue, with previous reports showing that the highest incidence were long bones^14^,^15^,^16^,^17^, but with the change of the PBL definition, multiple sites of bone invasion have also been included in the scope of PBL. After that, there are data suggesting the most common diseased parts were the spine or pelvis^7^,^8^,^18^. Current treatments include surgery, radiotherapy and chemotherapy, but there is no standard treatment due to a lack of comparability among the studies, caused by changed PBL definitions.

Since PBL is a rare disease, current studies about PBL are mostly retrospective or case reports and retrospective studies often lasted over more than ten years or even decades^13^. In addition, present research results are mostly from the US and Europe, but data from Asia are limited. Here we retrospectively collected and analyzed data from 61 PBL patients admitted to our center from 1997 to 2014, in order to better understand the characteristics, outcome, and prognostic factors of PBL in Chinese population. To the authors’ knowledge, this study comprises the largest sample size analyzed in Asia currently.

## Patients and Methods

### Patients

In this retrospective study, 61 PBL patients from the department of lymphoma of Peking University Cancer Hospital and Institute from January 1997 to January 2014 were included. All patients satisfied the 2013 WHO criteria of PBL^4^: lymphoma was restricted to bone and adjacent soft tissue with or without regional lymph node involvement at the time of diagnosis. Patients with lymph node involvement on the other side of the diaphragm, distant bone marrow involvement or any other site of extranodal disease were excluded in this series.

The study was approved by the Ethical committee of Peking University Cancer Hospital and Institute and written informed consent was provided by the patients. All experiments were performed in accordance with relevant guidelines and regulations.

## Methods

### Information Reviewed

The database was established from the medical records including: gender, age, presenting symptoms, involved sites, Eastern Cooperative Oncology Group (ECOG) score^19^, radiological findings, pathological diagnosis, stage, international prognostic index (IPI)^20^, treatment modality and treatment response. All patients were followed up by outpatient reviews or by telephone conversations; the last follow-up date was 2015-1-1. Overall survival (OS) was defined as time from pathological diagnosis until death, lost or last follow-up. Progression free survival (PFS) was defined as time from pathological diagnosis until disease progression, lost or last follow-up.

### Staging

All patients underwent detailed history and physical examinations, blood tests, imaging (chest X-ray or computer tomography (CT), abdominal B-scan ultrasonography or CT, systemic superficial lymph node B-scan ultrasonography; some patients received a systemic positron emission tomography (PET)/CT examination) as well as bone marrow aspirate and biopsy. Some of the patients received a cerebrospinal fluid examination when the spine was involved. The clinical stage was determined by Ann Arbor staging criteria^21^. Stage IE was defined as a solitary bone lesion without lymph node involvement; stage IIE as a solitary bone lesion with regional lymph nodes involvement; and stage IV was the presence of multiple bone lesions with or without regional lymph node involvement.

### Response Criteria

Response to treatment was assessed by the International Workshop to Standardize Response Criteria in 1999 (IWC), also known as the Cheson criteria^22^. The PET/CT review efficiency of some patients was based on the revised edition of malignant lymphoma remission criteria in 2007^23^.

### Statistical methods

All data were statistically analyzed by SPSS Statistics for Windows (Version 22.0. Armonk, NY: IBM Corp.). The Kaplan-Meier method was used for survival analysis, and the Log-rank test to analyze the survival rate between the two groups. Variables achieving significant level of P < 0.05 were entered into the COX proportional hazards regression model to complete multivariable analyses. Independent prognostic factors were determined if they had significant effect in the Cox model (P < 0.05).

## Results

### Clinical features

The general clinical characteristics of 61 patients are shown in Table 1. The proportion of males to females of 61 patients was 1.65:1. The median age was 45 years (range, 13–80 years). The most common initial symptom was local pain, followed by nerve compression and local mass.

The histopathological subtypes of the 61 PBL patients are shown in Table 2. DLBCL was the most common histological type, accounting for 55.7% (34 cases), followed by systemic anaplastic large cell lymphoma (ALCL) and B-lymphoblastic lymphoma (B-LBL), 13.1% (8 cases) and 11.5% (7 cases), respectively. Other rare types included: Hodgkin’s lymphoma (HL), mantle cell lymphoma (MCL), marginal zone B-cell lymphoma (MZL), T-lymphoblastic lymphoma (T-LBL), as well as two cases of T-cell origin (failed to be classified). T-cell lymphoma accounted for 18.0% of the cases.

The pathogenic sites of the 61 PBL patients are shown in Table 3. For single bone invasion, the most common site was pelvic bone (9 cases, 33.3%), followed by the long bone with a total of 8 cases (29.6%). In patients with multiple bone invasions, the most common site was the spine (25 cases, 73.5%), followed by the pelvic bones (17 cases, 50%). In all patients, the incidence of the most common sites were the spine and pelvis bones, followed by the skull, femur and humerus.

### Treatments, responses and survival of patients with PBL

The treatment modality that 61 PBL patients received are shown in Table 4. All patients underwent initial therapy with systemic chemotherapy, of which 37 cases (60.7%) received chemotherapy alone and 24 patients (39.3%) were treated with combined local radiotherapy. 18 patients underwent surgery in which the purpose for 10 patients was to ease spinal cord compression or treat pathological fractures and 8 patients underwent primary lesion resections.

After the initial treatment that all patients completed, 57 patients’ clinical data can be evaluated. 32 patients achieved complete remission (CR), 18 patients obtained partial remission (PR) and the overall response rate (ORR) was 87.7% (56.1% CR + 31.6% PR). 3 cases were assessed as stable disease (SD) and 4 cases as progress disease (PD) after initial treatment.

**Figure 1:**
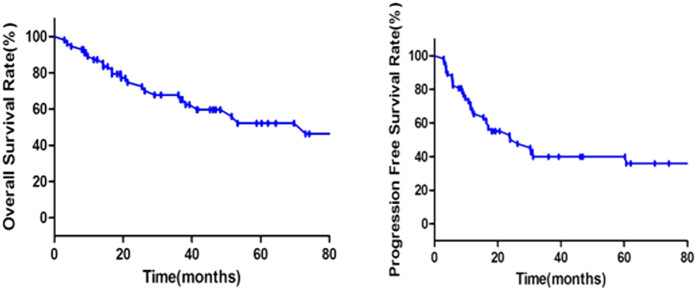
Overall survival and progression-free survival of 53 PBL patients.

A survival analysis was done for 57 PBL patients with complete follow-up data. The median follow-up was 31 months (range, 3–216 months). By the date of the last follow-up, 35 patients survived (61.4%), 22 deaths occurred (38.6%), of which 20 patients died of tumor progression and 2 died due to the treatment. The 5-year OS was 52.3% with the 5-year PFS being 40.1% (Fig. 1).

### Effect of the histopathological subtypes on the prognosis of patients with PBL

In this group of patients, in addition to DLBCL, other pathology samples were few and thus the survival among all histopathological subtypes could not be compared. The histopathological subtypes B-cell lymphoma, T-cell lymphoma and Hodgkin’s lymphoma showed no significant difference for the survival among this three groups. There was also no significant difference in the survival between patients with DLBCL and non-DLBCL. In addition, no significant difference was found for OS and PFS between ALCL and non-ALCL patients, or between DLBCL patients and ALCL patients (Table 5).

### Analysis of Survival and prognostic factors in patients with PB-DLBCL

**Figure 2:**
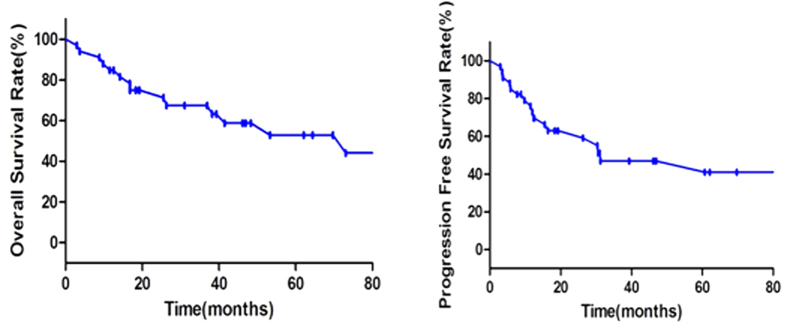
Overall survival and progression-free survival of 34 PB-DLBCL patients.

Given the effects of different histopathological subtypes on survival and the sample size limitation of other pathological types, we only analyzed prognostic factors of 34 DLBCL patients. The median follow-up was 38 months (range, 3–216 months). The 5-year OS was 53.0%, 5-year PFS was 47.0% (Fig. 2).

**Figure 3:**
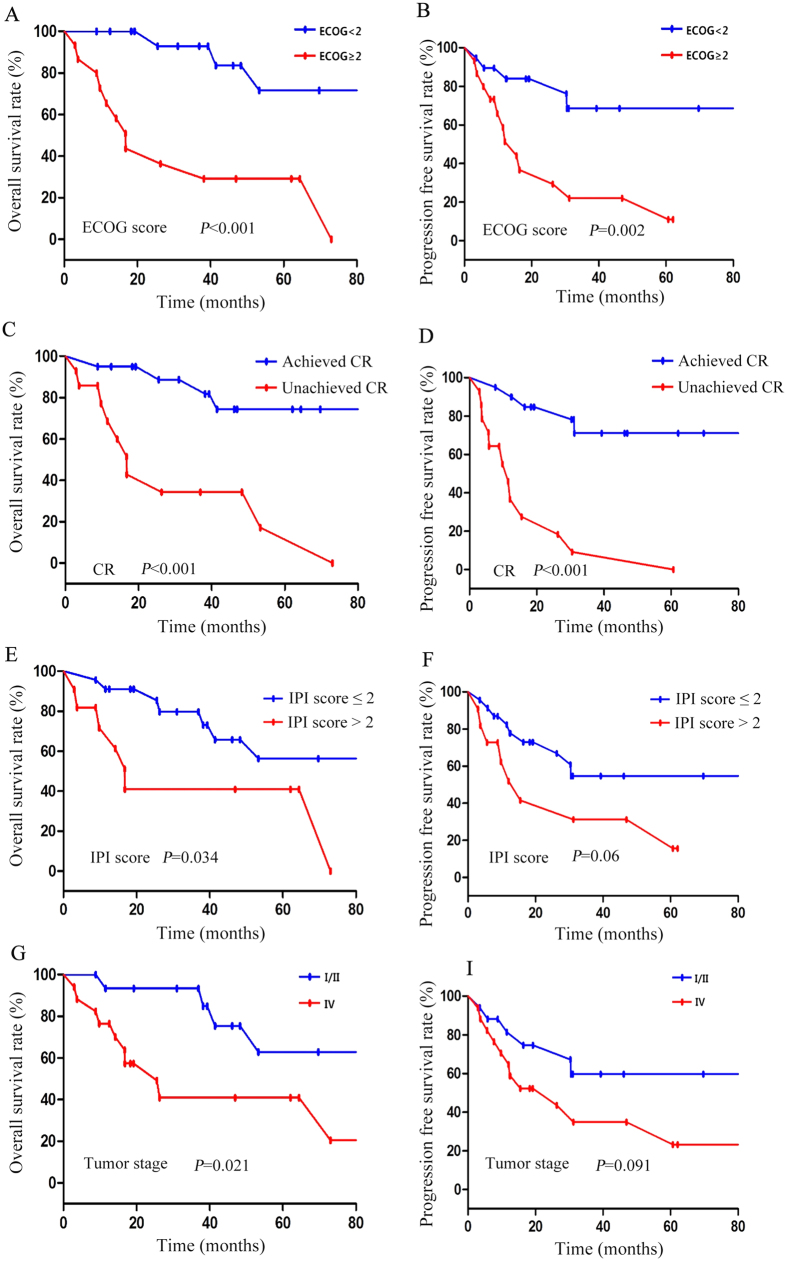
Univariable analyses of prognostic factors for overall survival (OS) and progression-free survival (PFS) in patients with PB-DLBCL. (**A**) OS according to ECOG score. (**B**) PFS according to ECOG score. (**C**) OS according to respose to initial therapy. (**D**) PFS according to respose to initial therapy. (**E**) OS according to International Prognostic Index. (**F**) PFS according to International Prognostic Index. (**G**) OS according to stage. (**H**) PFS according to stage.

**Figure 4:**
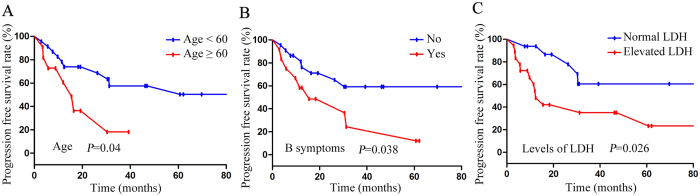
Univariable analyses of prognostic factors for progression-free survival (PFS) in patients with PB-DLBCL. (**A**) PFS according to age. (**B**) PFS according to B symptoms. (**C**) PFS according to LDH levels.

The Kaplan-Meier method and log-rank test was used to analyze the following factors for univariable analysis: gender, age, pathogenic sites, pathological fractures, B symptoms, ECOG score, LDH levels, soft tissue invasion, lymph node involvement, stage, IPI score, molecular subtypes (germinal center B-cell-like (GCB) vs. non-GCB), treatment modality, response to initial therapy and rituximab use. High ECOG score (≥2), stage IV (stage IV vs. stage I&II), high IPI score (>2) and unachieved CR in initial therapy were associated with worse OS (Fig. 3 and Table 6), whereas older age (≥60), B symptoms, high ECOG score (≥2), elevated LDH and unachieved CR in initial therapy were associated with worse PFS (Figs 3, 4 and Table 6).

23 cases out of a total of 34 PB-DLBCL patients were further divided into GCB type (11 cases) and non-GCB type (12 cases), with 5-year OS rates of the 2 groups being 70.1% and 20.0% respectively, but the apparent difference did not reach statistical significance (*P* = 0.193) and also for PFS there was no statistical significance (*P* = 0.299). 20 patients received combined rituximab treatments, but compared with the group without rituximab, OS and PFS were not significantly different (5-year OS 48.4% *vs* 60.6%, *P* = 0.494; 5-year PFS 44.9% *vs* 44.0%, *P* = 0.432). The OS rate of patients with complicated pathologic fractures appeared to be inferior to patients without pathological fractures, but the trend did not reach statistical significance (5-year OS 31.7% *vs* 58.9%, *P* = 0.066) and also PFS rates did not significantly differ between the two groups (*P* = 0.240) (Table 6).

Multivariable analysis using a COX proportional hazards regression model showed that the baseline ECOG score and response to initial treatment were independent factors for the OS of PB-DLBCL patients. Response to initial treatment was also an independent risk factor for PFS of the patients (Table 7).

## Discussion

In previous studies, the definition, clinical characteristics, treatment modalities, and prognosis of PBL remain controversial and most of the present research results are from the US and Europe. In this report, we described a series of Chinese PBL patients using the new 2013 WHO criteria^4^. This study comprises the largest sample size analyzed in Asia.

According to previous studies, the majority of PBL belongs to B-cell NHL, the most common type being DLBCL, occurring in about ≥80% of all cases, followed by follicular lymphoma^7^,^8^,^9^ while T-cell NHL accounts for about 1–5% of all PBL patients in the US and Europe^7^,^8^,^14^,^24^. In our study, although DLBCL was the most common histological type (55.7%), the incidence seems to be lower than previous reports in the US and Europe. T-cell lymphoma accounted for 18.0% of all PBL patients in our study. The incidence of T-cell lymphoma was higher than previous reports in the US and Europe but similar to other Asia series^18^,^25^,^26^. We believe that this differences are due to regional differences of T-cell lymphoma incidences being higher in Asia than in the US and Europe^27^.

Most previous studies suggested a predominance of long bone involvement in PBL^14^,^15^,^16^,^17^. However, Ramadan *et al.*^8^ reported that the spine was the most commonly involved site, accounting for one-third of 131 cases. Others studies from China and Japan showed that the pelvis was the most common site of PLB involvement^18^,^26^,^28^. In the present study, the most commonly involved sites were the spine and pelvis (both accounting for 42.6%, respectively). The preponderance of pelvis involvement may be a unique characteristic of Asian patients with PBL.

The overall outcome of PBL is controversial. In our study, the overall 5-year OS and PFS of 57 PBL patients was 52.3% and 40.5% and the 5-year OS and PFS of 34 PB-DLBCL patients among them were 54.7% and 49.1%. According to previous reports, 5-year OS of PBL patients were 88%^15^, 76%^7^, 57.8%^29^ and 55%^18^. However, although DLBCL accounts for a large proportion (68–83%), these studies did not exclude the effect of histological heterogeneity on survival of PBL; we therefore consider that these data lack comparability. To exclude the effect of different histological type on prognosis, fewer studies have discussed the prognosis of PB-DLBCL alone and suggested that PB-DLBCL has a better prognosis than other types of DLBCL. Wu *et al.*^9^ reported the 5-year OS of 53 PB-DLBCL cases was 81.1%. Small sample data from India^30^ showed an 8-year OS and DFS of 21 PB-DLBCL patients of 95.2% and 100%. Heyning *et al.*^31^ reported that in a group of 36 PB-DLBCL cases from the Netherlands, the 5-year OS was 75%. However, our study did not suggest such a good prognosis of PB-DLBCL, as reported by some other authors. Jawad *et al.*^24^ reported the 5-year and 10-year OS of 994 PB-DLBCL cases were 61% and 48%, respectively. Ramadan *et al.*^8^ reported on 131 PBL patients from which the 5-year and 10-year OS of 103 (79%) PB-DLBCL patients were 62% and 41%, respectively. Considering variations in the definition and the treatment of PBL and selection bias in retrospective studies, it is perhaps not surprising that there were quite different outcomes between independent studies.

The prognostic factors of PBL have not been well established. In nodal lymphoma, pathological type is one of the most important prognostic factors. Superior prognosis of PB-DLBCL compared to non-DLBCL patients has been noted by Hsieh *et al.*^25^ who documented 14 cases of PBL in Taiwan and concluded that the prognosis of B-cell PBL was better than T-cell PBL (P = 0.016). Other studies also reported histological type to be a prognostic factor^16^,^32^. However, in our series of PBL patients including B-cell lymphoma, T-cell lymphoma, and Hodgkin’s lymphoma, no statistical difference in prognosis was found between the three groups. And neither DLBCL group nor ALCL group showed a superior prognosis when compared to other pathological subtypes. Due to the small sample size, it was not possible to compare further differences in prognosis between the various pathological types. Thus, we believe that the impact of pathological type on prognosis of PBL remains an open question.

To exclude the effect of the pathological type on prognosis, in our study we used univariable and multivariable analyses only on PB-DLBCL patients. The IPI system was developed to assess prognosis in patients with aggressive NHL. High IPI score had been considered as a poor prognostic factor of PBL by Ramadan *et al.*^8^ , Wu *et al.*^9^ and Huang *et al.*^18^, but not by Catlett *et al.*^33^ and Alencar *et al.*^14^. In the present study, IPI and its variants(age, ECOG score, LDH levels, number of extranodal sites and Ann Arbor stage) were analyzed. Univariable analyses showed that IPI score, tumor stage and ECOG score had a significant impact on prognosis, but only the ECOG score was identified to be an independent prognostic factor in multivariable Cox analysis. The prognostic impact of IPI on patients with PLB still requires further discussion. Some studies have suggested that age was an important factor affecting the prognosis of PBL^8^,^15^,^16^,^34^,^35^. In our study, although age had an impact on the PFS of PB-DLBCL patients, there was no significant effect of age on OS rates and also multivariable analysis showed no age effect. In addition to ECOG score, whether CR in initial treatment was an independent prognostic factor determining both OS and PFS, which was consistent with some previous reports^7^,^36^. A previous study aimed at nodal DLBCL, suggested that the prognosis of the GCB subtype is better than that of the non-GCB subtype in using standard chemotherapy^37^. In our study, 23 cases of 34 PB-DLBCL patients could be further divided into GCB type (11 cases) and non-GCB type (12 cases), but the 5-year OS and PFS between the 2 groups did not reach statistical significance, which is in accordance with reports from Bhagavathi *et al.*^38^ and Heyning *et al.*^31^, but the sample sizes were small and the influence of molecular subtypes on prognosis remains to be elucidated.

Treatment modalities for PBL include chemotherapy, radiotherapy and surgery which is mainly applied for diagnostic biopsy, to repair pathologic fractures or for spinal cord compression therapies. In our PB-DLBCL group of patients, the prognosis of patients accepting excisions was not better than the prognosis of patients who did not accept excisions and chemotherapy was the main form of treatment for the PBL patients. Various studies have noted that combined modality therapy (CMT) was better than radio/chemotherapy alone for PBL^7^,^11^,^15^,^17^. However, there is still controversy in whether CMT is superior to chemotherapy alone. Cai *et al.*^7^ reported 116 early PBL cases, with 5-year OS rates of 79% for the CMT group and 69% for the radio/chemotherapy alone groups (P = 0.05) and a multivariable analysis showed that CMT was an independent factor that affects OS. Report by Beal *et al.*^15^ also revealed that CMT was an independent prognostic risk factor for PBL, with 5-year OS rate of 95% for the CMT group and 78% for the solely radio/chemotherapy groups (P = 0.001). In our study, all of 34 PB-DLBCL patients received chemotherapy and 18 patients (42.1%) received CMT. However, the addition of radiotherapy to chemotherapy did not improve the prognosis of PB-DLBCL in our study. Similar data were also reported in a study by Alencar *et al.*^14^ and Ramadan *et al.*^8^ Taken together we believe, regardless of the stage at diagnosis, PBL should still be regarded as a systemic disease like other lymphomas, with systemic chemotherapy being the main treatment. Combined radiotherapy in the present study failed to improve the prognosis of PBL patients, but because of the limited sample size and the results of previous studies, the role of radiotherapy still needs further verification. Whether PBL patients need CMT, the treatment modality should be selected in the clinic individually according to the actual condition of the patient. However, we suggest that surgical resection of the lesion is not appropriate as a preferred treatment for PBL patients.

Rituximab in combination with chemotherapy has been used as standard protocol for CD20 + B-cell non-Hodgkin’s lymphoma. The vast majority of the pathological types of PBL are B-cell related, but whether the addition of rituximab can improve the prognosis of PBL patients remains controversial. Ramadan *et al.*^8^ compared the prognosis between PB-DLBCL patients receiving CHOP/CHOP-like chemotherapy and those receiving an R-CHOP program, and found that rituximab significantly improved PFS. In the report of Alencar *et al.*,^14^ the trend of prolonged PFS has been apparently improved in PB-DLBCL patients receiving rituximab-CHOP compared to those on a CHOP regime alone, but statistical significance (P = 0.062) was not reached. Also Catlett *et al.*^33^ and Kim *et al.*^39^ reported non-significant trend towards improved OS with rituximab combination therapy, which is in agreement with our result. Thus, the role of rituximab in PBL treatments requires further investigation.

## Conclusion

By retrospective analysis 61 PBL patients in our single institution, we identified the clinical characteristics and prognosis of PBL in Chinese population. The results showed that the most common pathological type was DLBCL, but the proportion of the T-cell type cases was higher than in the US and Europe. The most common sites invaded were the bones of the spine and the pelvis. High baseline ECOG scores and unachieved CR in initial therapy result in poor prognosis of PB-DLBCL patients. Chemotherapy plays a central role in the treatment of PBL. Though the present result failed to support the use of combined modality for the treatment of PBL, the role of radiotherapy and optimal treatment strategy for PBL warrants further investigation by larger prospective multicenter studies.

## Additional Information

**How to cite this article**: Zhang, X.Y. *et al.* Clinical characterization and outcome of primary bone lymphoma: a retrospective study of 61 Chinese patients. *Sci. Rep.* **6**, 28834; doi: 10.1038/srep28834 (2016).

## Figures and Tables

**Table 1:** General clinical characteristics of 61 patients with PBL.

Item	Cases	Proportion (%)
Gender		
Male	38	62.3
Female	23	37.7
Female: Male	1.65: 1	
Age		
<60	46	75.4
≥60	15	24.6
Median age	45 (13–80)	
Initial symptom		
Pain	50	82.0
Nerve compression	20	32.8
Local mass	7	11.5
B symptoms	19	31.1
Pathological fracture	13	21.3
Soft tissue invasion	42	68.9
Lymph node involvement	27	44.3
The number of lesions		
Single	28	45.9
Multiple	33	54.1
Stage		
Stage IE	16	26.2
Stage IIE	11	18.1
Stage IV	34	55.7
ECOG score		
≤2	37	60.7
>2	24	39.3
LDH		
Normal	32	52.5
Elevated	29	47.5
IPI score		
≤2	44	72.1
>2	17	27.9

ECOG, Eastern Cooperative Oncology Group; LDH, lactate dehydrogenase; IPI, International Prognostic Index.

**Table 2:** Histopathological subtypes of 61 PBL patients.

Pathological type	Cases	Proportion (%)
DLBCL	34	55.7
GCB type	11	18.0
Non-GCB type	12	19.7
Undetected	11	18.0
ALCL	8	13.1
ALK(+)	5	8.2
ALK(−)	3	4.9
B-LBL	7	11.5
MCL	3	4.9
MZL	2	3.3
T-LBL	1	1.6
HL	4	6.6
T-cell origin	2	3.3

DLBCL, diffuse large B-cell lymphoma; GCB, germinal center B-cell-like; Non-GCB, non-germinal center B-cell-like; ALCL, anaplastic large cell lymphoma; ALK, anaplastic lymphoma kinase; B-LBL, B-lymphoblastic lymphoma; MCL, mantle cell lymphoma; MZL, marginal zone lymphoma; T-LBL, T-lymphoblastic lymphoma; HL, Hodgkin lymphoma.

**Table 3:** Pathogenic sites of 61 PBL patients.

Site	Cases (%)	Multiple	Total
	Single		
Limbs			
Humerus	3 (11.1)	4 (11.8)	7 (11.5)
Femur	2 (7.4)	6 (17.6)	8 (13.1)
Tibia, fibula	3 (11.1)	2 (5.9)	5 (8.2)
Axial skeleton			
Spine	1 (3.7)	25 (73.5)	26 (42.6)
Pelvis	9 (33.3)	17 (50.0)	26 (42.6)
Rib cage	0 (0)	5 (14.7)	5 (8.2)
Shoulder blade	2 (7.4)	2 (5.9)	4 (6.6)
Clavicle	0 (0)	2 (5.9)	2 (3.3)
Skull	7 (25.9)	6 (17.6)	13 (21.3)
Total	27 (100.0)	34 (100.0)	61 (100.0)

**Table 4:** Treatment modality of 61 PBL patients.

Treatment	Cases	Proportion (%)
Chemotherapy	37	60.7
Chemotherapy and Radiotherapy	24	39.3
Surgery	18	29.5
Relieve symptoms or treat fractures	10	16.4
Resect primary lesions	8	13.1

**Table 5:** Effect of histopathological subtypes on the prognosis of patients with PBL.

Item	Cases	OS			PFS		
		5-year (%)	95% CI	*P*-value	5-year (%)	95% CI	*P*-value
Pathological types							
B cell lymphoma	43	52.1	42.9–61.3	0.771	43.8	35.3–52.3	0.207
T cell lymphoma	10	44.4	23.3–65.5		45.0	27.6–62.4	
HL	4	75.0	53.3–96.7		0.0		
Whether to be DLBCL							
DLBCL	34	53.0	42.8–63.2	0.805	47.0	37.6–56.4	0.184
Not DLBCL	23	51.2	37.6–64.8		28.7	17.6–39.8	
Whether to be ALCL							
ALCL	8	75.0	59.7–90.3	0.667	62.5	45.4–80.2	0.633
Not ALCL	49	49.9	41.2–58.6		37.0	29.3–44.7	
DLBCL vs. ALCL							
DLBCL	34	53.0	42.8–63.2	0.680	47.0	37.6–56.4	0.907
ALCL	8	75.0	59.7–90.3		62.5	45.4–80.2	

OS, overall survival; PFS, progression-free survival; CI, confidence interval; HL, Hodgkin lymphoma; DLBCL, diffuse large B-cell lymphoma; ALCL, anaplastic large cell lymphoma.

P-values are shown for the log-rank test between variables.

**Table 6:** Univariable analysis of 34 PB-DLBCL patients.

Item	Cases	OS			PFS		
		5-year (%)	95% CI	*P-*value	5-year (%)	95% CI	*P-*value
Gender							
Male	20	57.7	45.1–70.3	0.980	47.9	35.1–60.7	0.805
Female	14	43.3	26.5–60.1		46.4	32.6–60.2	
Age							
<60	23	60.9	49.7–72.1	0.288	57.6	46.6–68.6	0.040
≥60	11	25.6	5.2–46.0		18.2	3.0–33.4	
Pathogenic sites							
Axial skeleton	19	63.9	51.7–76.1	0.827	58.3	45.8–70.8	0.366
Limbs	15	39.3	23.5–55.1		36.7	23.8–49.6	
Pathological fracture							
Yes	9	31.7	13.7–49.7	0.066	33.9	15.7–52.1	0.240
No	25	58.9	46.8–71.0		50.9	40.0–61.8	
B symptoms							
Yes	12	42.9	26.4–29.4	0.084	24.3	10.1–38.5	0.038
No	22	61.1	49.3–72.9		59.3	48.0–70.6	
ECOG score							
<2	19	71.6	57.2–86.0	<0.001	68.6	56.6–80.6	0.002
≥2	15	29.1	16.9–41.3		22.0	10.8–33.2	
LDH							
Normal	16	54.1	38.4–69.8	0.185	60.6	46.6–74.6	0.026
Elevated	18	52.7	40.4–65.0		35.1	23.3–47.0	
Soft tissue invasion							
No	7	57.1	32.2–82.0	0.360	57.1	32.2–82.0	0.179
Yes	27	44.9	33.4–56.4		37.1	26.8–47.4	
Lymph node invasion							
No	14	68.8	55.5–82.1	0.465	55.0	38.7–71.3	0.121
Yes	20	42.4	28.7–56.1		30.9	19.2–42.6	
Stage							
Stage I & II	17	62.9	47.4–78.4	0.021	59.7	46.8–72.6	0.091
Stage IV	17	41.0	27.9–54.1		34.9	21.9–47.9	
IPI score							
≤2	23	56.4	43.1–69.7	0.034	54.7	43.1–66.3	0.060
>2	11	40.9	25.3–56.5		31.2	16.4–46.0	
Molecular subtype							
GCB	11	70.1	55.4–84.8	0.193	63.6	49.1–78.1	0.299
Non-GCB	12	20.0	2.9–37.1		20.8	4.1–37.5	
Undetected	11						
Radiotherapy							
Yes	18	57.4	44.8–70.0	0.423	43.8	31.9–55.7	0.684
No	16	49.2	32.1–66.3		52.5	37.0–68.0	
Prophylactic intrathecal chemotherapy							
Yes	10	75.0	59.2–90.8	0.560	63.0	45.3–80.7	0.472
No	24	44.9	32.9–56.9		41.6	30.9–52.3	
Surgical resection							
Yes	4	50.0	14.6–85.4	0.457	50.0	14.6–85.4	0.323
No	30	54.1	44.1–64.1		45.0	35.2–54.8	
Whether CR in initial therapy							
Yes	20	74.4	63.1–85.7	<0.001	71.1	59.9–82.3	<0.001
No	14	17.1	3.2–31.0		9.2	0.5–17.9	
Rituximab							
Used	21	48.4	34.6–62.2	0.494	44.9	32.3–57.5	0.432
Unused	13	60.6	46.8–74.4		44.0	29.7–5	

OS, overall survival; PFS, progression-free survival; CI, confidence interval; ECOG, Eastern Cooperative Oncology Group; LDH, lactate dehydrogenase; IPI, International Prognostic Index; GCB, germinal center B-cell-like; Non-GCB, non-germinal center B-cell-like; CR, complete remission.

P-values are shown for the log-rank test between variables.

**Table 7:** Multivariable analysis on the effect to survival of PB-DLBCL patients.

Risk factors	OS			PFS		
	RR	95% CI	*P*	RR	95% CI	*P*
ECOG score (ECOG ≥2 *vs.* ECOG <2)	4.840	1.261–18.578	0.022			NS
Response to initial treatment (CR *vs.* no CR)	0.245	0.071–0.850	0.027	0.112	0.038–0.332	0.001

RR, Relative risk; CI, confidence interval; NS, No statistical significance; ECOG, Eastern Cooperative Oncology Group; CR, complete remission.
